# PPARG c.1347C>T polymorphism is associated with cancer susceptibility: from a case-control study to a meta-analysis

**DOI:** 10.18632/oncotarget.20925

**Published:** 2017-09-15

**Authors:** Hao Ding, Yuanmei Chen, Hao Qiu, Chao Liu, Yafeng Wang, Mingqiang Kang, Weifeng Tang

**Affiliations:** ^1^ Department of Respiratory Disease, Affiliated People's Hospital of Jiangsu University, Zhenjiang, Jiangsu Province, China; ^2^ Department of Thoracic Surgery, Fujian Cancer Hospital, Fujian Medical University Cancer Hospital, Fuzhou, Fujian Province, China; ^3^ Department of Immunology, School of Medicine, Jiangsu University, Zhenjiang, Jiangsu Province, China; ^4^ Department of Cardiothoracic Surgery, Affiliated People's Hospital of Jiangsu University, Zhenjiang, Jiangsu Province, China; ^5^ Department of Cardiology, The People's Hospital of Xishuangbanna Dai Autonomous Prefecture, Jinghong, Yunnan Province, China; ^6^ Department of Thoracic Surgery, Fujian Medical University Union Hospital, Fuzhou, Fujian Province, China

**Keywords:** PPARG, polymorphism, non-small cell lung cancer, risk

## Abstract

Recently, several studies suggested that *PPARG c.1347C>T* polymorphism was correlated with cancer risk. However, past results remained controversial. In this study, we performed a case-control study on the relationship of *PPARG c.1347C>T* polymorphism with risk of non-small cell lung cancer (NSCLC) and subsequently carried out a meta-analysis to further assess the association between *PPARG c.1347C>T* and overall cancer. In our case-control study, after adjusting by age, sex, body mass index (BMI), smoking and drinking, a tendency to increased NSCLC risk was noted (CT/TT vs. CC: adjusted OR, 1.21; 95% CI, 0.97–1.51; *P* = 0.097). In the meta-analysis, we found a significant association between *PPARG c.1347C>T* polymorphism and overall cancer risk (T vs. C: OR, 1.13; 95% CI, 1.03–1.23; *P* = 0.006; TT vs. CC: OR, 1.29; 95% CI, 1.07–1.56; *P* = 0.008, CT/TT vs. CC: OR, 1.11; 95% CI, 1.02–1.21; *P* = 0.014 and TT vs. CT/CC: OR, 1.26; 95% CI, 1.04–1.52; *P* = 0.016). In a subgroup analysis by ethnicity, evidence of significant association between *PPARG c.1347C>T* polymorphism and cancer risk was found among Asians and mixed populations. In a subgroup analysis by cancer type, *PPARG c.1347C>T* polymorphism was associated with risk of esophageal cancer and glioblastoma. In addition, in a subgroup analysis by origin of cancer cell, evidence of significant association between *PPARG c.1347C>T* polymorphism and cancer risk was also found among epithelial tumor. In conclusion, the findings indicate *PPARG c.1347C>T* polymorphism may increase the susceptibility of cancer.

## INTRODUCTION

It is reported that about 14.1 million cancer patients and 8.2 million cancer-related deaths have occurred in 2012 worldwide [1]. In developing countries, the survival of cancer is poorer compared with the developed countries. The possible reason of this phenomenon is most likely due to limited access and lack of standard treatment. Cancer burden could be decreased through the application of tobacco control, healthier dietary intake, vaccine injection, early detection and treatment, and so on [2]. It is thought that cancer results from the interaction of individual's genetic components with environmental factors [3].

Peroxisome proliferator-activated receptor gamma (PPARG) involves three isoforms (e.g. PPARG1, PPARG2, and PPARG3). PPARG is an important nuclear receptor which acts as a transcriptional regulator and regulates energy metabolism [4]. In the pathological process of obesity, insulin insufficient/resistance and diabetes, PPARG may be activated, and then promotes the accumulation of fatty tissue [5]. PPARG agonists enhance insulin sensitivity [6]. PPARG may also possess anti-inflammatory roles [7, 8]. Activation of PPARG could inhibit the production of many cytokines [e.g. tumor necrosis factor-alpha, interleukin (IL)-6, and IL-8] by antagonizing the activities of the signal transducer and activator of transcription, transcription factors activator protein 1, and nuclear factor-kappa-B, which inhibits the induction of inflammatory response [9]. A number of case-control studies demonstrated that obesity, insulin resistance/insufficient, metabolic syndrome and inflammation were correlative conditions in which PPARG could modify and regulate these actions, and influence the risk of cancer [10–12].

Recently, a number of studies focused on the association of *PPARG* polymorphisms with cancer risk [13–28]. *PPARG* NM_015869.4:c.34C>G (rs1801282 C>G) and NM_138712.3: c.1347C>T (rs3856806 C>T) polymorphisms are two common single nucleotide polymorphisms (SNPs). A meta-analysis indicated the *PPARG* c.34C>G polymorphism was associated with the risk of cancer in Asians [29]. However, the association of *PPARG* c.1347C>T polymorphism with cancer risk was not found. Several meta-analyses did not identify the association between this SNP and cancer risk [30, 31]. Although more and more case-control studies focused on the relationship of the *PPARG* c.1347C>T polymorphism with cancer susceptibility, the obtained findings remained conflicting. In addition, the association between this polymorphism and lung cancer was not studied in Asians. Therefore, in this study, we designed a case-control study and assessed the relationship between *PPARG* c.1347C>T polymorphism and risk of non-small cell lung cancer (NSCLC) in Eastern Chinese Han population. Meta-analysis is a useful method of promoting the effective sample size by pooling of individual data from the enrolled studies, thus strengthening the power of the study for the assessment of genetic effects [32]. To address the association between *PPARG* c.1347C>T polymorphism and cancer risk more precisely, we carried out a comprehensive meta-analysis.

## RESULTS

### Association of *PPARG* c.1347C>T polymorphism with NSCLC

The risk factors, anthropometric data as well as demographics are listed in Table 1. Body mass index (BMI) of controls was significantly higher than it in NSCLC group (*P* < 0.001). This study was well-matched by age and gender. The SNP information of *PPARG* c.1347C>T is shown in Table 2. The genotyping success rate was 99.94% in 1,551 samples. Table 2 summarizes the minor allele frequency (MAF) of *PPARG* c.1347C>T polymorphism and Hardy-Weinberg Equilibrium (HWE) in controls.

**Table 1:** Distribution of selected demographic variables and risk factors in NSCLC cases and controls

Variable	Overall Cases (*n* = 521)	Overall Controls (*n* = 1,030)	*P*^a^
	*n* (%)	*n* (%)	
Age (years)	59.76 ±10.71	60.34 ±9.11	0.268
Age (years)			0.843
< 60	238 (45.68)	476 (46.21)	
≥ 60	283 (54.32)	554 (53.79)	
Sex			0.453
Male	287 (55.09)	588 (57.09)	
Female	234 (44.91)	442 (42.91)	
Smoking status			**< 0.001**
Never	317 (60.84)	828 (80.39)	
Ever	204 (39.16)	202 (19.61)	
Alcohol use			**< 0.001**
Never	444 (85.22)	949 (92.14)	
Ever	77 (14.78)	81 (7.86)	
BMI (kg/m^2^)	23.00 (±3.03)	23.84 (±3.06)	**< 0.001**
BMI (kg/m^2^)			
< 24	337 (64.68)	547 (53.11)	**< 0.001**
≥ 24	184 (35.32)	483 (46.89)	

^a^Two-sided *χ*^2^ test and Student t test

BMI: body mass index

**Table 2:** Primary information for PPARG c.1347C>T polymorphism

Genotyped SNPs	PPARG c.1347C>T
Chromosome	3
Function	coding-synonymous
Chr Pos (NCBI Build 37)	12475557
MAF^a^for Chinese in database	0.25
MAF in our controls (*n* = 1,030)	0.21
*P* value for HWE^b^test in our controls	0.431
Genotyping method	SNPscan
% Genotyping value	99.94%

^a^MAF: minor allele frequency.

^b^HWE: Hardy–Weinberg equilibrium.

The frequencies of *PPARG* c.1347 CC, CT and TT genotypes were 57.01%, 38.00% and 4.99% in 521 NSCLC patients and 61.32%, 34.50%, and 4.18% in 1,030 non-cancer controls, respectively. The genotype distribution of *PPARG* c.1347C>T polymorphism is listed in Table 3. In controls, the genotype distribution of this polymorphism was in accord with HWE. When compared with the frequency of c.1347 CC genotype, the frequency of c.1347 CT genotype was not difference between the NSCLC patients and controls (crude OR = 1.19, 95% CI: 0.95–1.48, *P* = 0.130). When compared with the frequency of c.1347 CC genotype, there was also no difference in the frequency of c.1347 TT genotype between the NSCLC patients and the controls (crude OR = 1.29, 95% CI: 0.78–2.13, *P* = 0.329). When c.1347 CC genotype was used as reference, there was also no difference in the frequency of c.1347 TT/CT genotype between the NSCLC patients and the controls (crude OR = 1.20, 95% CI: 0.97–1.48, *P* = 0.102). In addition, When c.1347 CC/CT genotype was used as reference, we found that there was no difference in the frequency of c.1347 TT genotype between the NSCLC patients and the controls (crude OR = 1.20, 95% CI: 0.73–1.98, *P* = 0.465). Adjustments for age, sex, BMI, smoking and drinking, as demonstrated in Table 3, a tendency to increased NSCLC risk was noted (CT vs. CC: adjusted OR, 1.21; 95% CI, 0.96–1.53; *P* = 0.106; TT vs. CC: adjusted OR, 1.20; 95% CI, 0.71–2.04; *P* = 0.492, CT/TT vs. CC: adjusted OR, 1.21; 95% CI, 0.97–1.51; *P* = 0.097 and TT vs. CT/CC: adjusted OR, 1.12; 95% CI, 0.67–1.88; *P* = 0.671).

**Table 3:** Logistic regression analyses of associations between PPARG c.1347C>T polymorphism and risk of non-small cell lung cancer

Genotype	Cases (n = 521)		Controls (n = 1,030)		Crude OR (95%CI)	P	Adjusted OR^a^ (95%CI)	P
	n	%	n	%				
PPARG c.1347C>T								
CC	297	57.01	631	61.32	1.00		1.00	
CT	198	38.00	355	34.50	1.19 (0.95–1.48)	0.130	1.21 (0.96–1.53)	0.106
TT	26	4.99	43	4.18	1.29 (0.78–2.13)	0.329	1.20 (0.71–2.04)	0.492
CT+TT	224	42.99	398	38.68	1.20 (0.97–1.48)	0.102	1.21 (0.97–1.51)	0.097
CC+CT	495	95.01	986	95.82	1.00		1.00	
TT	26	4.99	43	4.18	1.20 (0.73–1.98)	0.465	1.12 (0.67–1.88)	0.671
T allele	250	23.99	441	21.43				

^a^Adjusted for age, sex, smoking status, alcohol use and BMI status.

### Meta-analysis of *PPARG* c.1347C>T polymorphism and cancer risk

**Figure 1:**
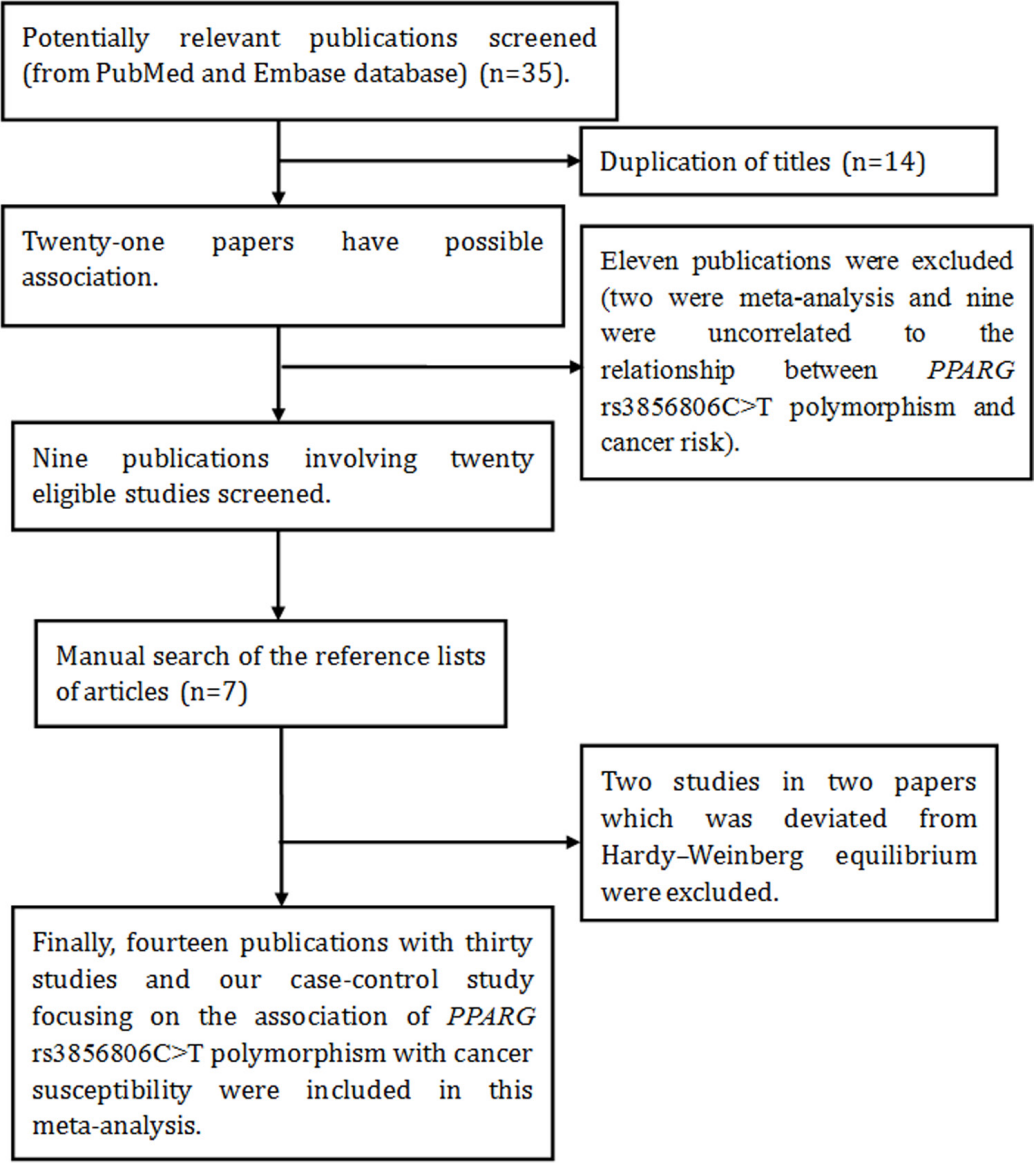
Flow diagram of the meta–analysis of the association between *PPARG* c.1347C>T polymorphism and cancer risk

Next, we carried out a pooled analysis to determine the potential relationship between *PPARG* c.1347C>T polymorphism and overall cancer risk. A total of 35 abstracts were retrieved from searching of EMBASE and Pubmed databases. The selecting process of literature is presented in Figure 1. In total, there were 14 publications [17, 21, 23, 24, 33–42] and our case-control study recruited in this meta-analysis. Some publications involved several subgroups [17, 21, 24, 33, 34, 38, 40, 42], we treated them separately. If 1 cancer type was studied by < 2 individual studies, then it was combined into the subgroup of ‘other cancers’. The characteristic of the included studies and *PPARG* c.1347C>T genotypes in different study are listed in Tables 4, 5. In total, 6,814 cases and 14,590 controls were enrolled in this meta-analysis.

**Table 4:** Characteristics of the studies in meta-analysis

Study	Publication year	Ethnicity	Country	Cancer type	The origin of cancer cell	Sample size (case/control)	Genotype method	Scores
Zhou et al. [40]	2000	Caucasians	USA	Glioblastoma	Non-epithelial tumor	52/80	DGGE	4
Zhou et al. [40]	2000	Caucasians	German	Glioblastoma	Non-epithelial tumor	44/60	DGGE	4
Smith et al. [34]	2001	Asians	Japan	bladder cancer	Epithelial tumor	31/27	DGGE	2
Smith et al. [34]	2001	Asians	Japan	cervical cancer	Epithelial tumor	20/27	DGGE	2
Smith et al. [34]	2001	mixed	USA	endometrial cancer	Epithelial tumor	69/80	DGGE	2
Smith et al. [34]	2001	Caucasians	UK	ovarian cancer	Epithelial tumor	31/65	DGGE	2
Smith et al. [34]	2001	Asians	Japan	ovarian cancer	Epithelial tumor	28/27	DGGE	2
Smith et al. [34]	2001	mixed	USA	ovarian cancer	Epithelial tumor	26/80	DGGE	2
Smith et al. [34]	2001	mixed	USA	prostate cancer	Epithelial tumor	38/80	DGGE	2
Smith et al. [34]	2001	Caucasians	UK	Renal cell carcinoma	Epithelial tumor	40/65	DGGE	2
Jiang et al. [21]	2005	Asians	India	colorectal cancer	Epithelial tumor	301/291	PCR-RFLP	7
Jiang et al. [21]	2005	Asians	India	colorectal cancer	Epithelial tumor	301/291	PCR-RFLP	7
Siezen et al.[24]	2006	Caucasians	The netherlands	colorectal cancer	Epithelial tumor	204/399	DNA sequence	8
Siezen et al. [24]	2006	Caucasians	The netherlands	colorectal cancer	Epithelial tumor	487/750	DNA sequence	8
Kuriki et al. [17]	2006	Asians	Japanese	colorectal cancer	Epithelial tumor	128/238	PCR-TCCP	8
Kuriki et al. [17]	2006	Asians	Japanese	colorectal cancer	Epithelial tumor	257/771	PCR-TCCP	7
Wang et al. [41]	2006	mixed	USA	lymphoma	Non-epithelial tumor	705/609	TaqMan	8
Vogel et al. [23]	2007	Caucasians	Denmark	colorectal cancer	Epithelial tumor	355/753	Not available	8
Mossner et al.[42]	2007	Caucasians	German	melanoma	Non-epithelial tumor	335/355	PCR-RFLP	7
Mossner et al. [42]	2007	Caucasians	German	melanoma	Non-epithelial tumor	497/435	PCR-RFLP	7
Chang et al.[33]	2008	Asians	China	ampulla of vater cancer	Epithelial tumor	47/786	TaqMan	7
Chang et al. [33]	2008	Asians	China	bile duct cancer	Epithelial tumor	127/786	TaqMan	7
Doecke et al.[38]	2008	mixed	Australia	esophageal cancer	Epithelial tumor	260/1352	sequencing	7
Doecke et al. [38]	2008	mixed	Australia	esophageal cancer	Epithelial tumor	301/1352	sequencing	7
Doecke et al. [38]	2008	mixed	Australia	esophageal cancer	Epithelial tumor	213/1352	sequencing	7
Chang et al. [33]	2008	Asians	China	gallbladder cancer	Epithelial tumor	237/786	TaqMan	7
Wu et al. [35]	2011	Asians	China	breast cancer	Epithelial tumor	291/589	RT-PCR	7
Wei et al. [37]	2013	Asians	China	breast cancer	Epithelial tumor	216/216	MALDI-TOF MS	3
Jeon et al. [39]	2013	Asians	China	gastric cancer	Epithelial tumor	196/397	TaqMan	7
Park et al. [36]	2014	Asians	Korea	breast cancer	Epithelial tumor	456/461	MALDI-TOF MS	6
Our study	2017	Asians	China	lung cancer	Epithelial tumor	521/1030	SNPscan	7

DGGE: denaturing gradient gel electrophoresis.

PCR-RFLP: polymerase chain reaction-restriction fragment length polymorphism.

PCR-CTPP: polymerase chain reaction with confronting two-pair primers.

RT-PCR: reverse transcription-polymerase chain reaction.

MALDI-TOF MS: Matrix-Assisted Laser Desorption/Ionization Time of Flight Mass Spectrometry.

**Table 5:** Distribution of *PPARG* c.1347C>T polymorphism genotype and allele

Study	Publication year	case			control			case		contraol		HWE
		CC	CT	TT	CC	CT	TT	T	C	T	C	
Zhou et al. [40]	2000	31	21	0	70	10	0	21	83	10	150	Yes
Zhou et al. [40]	2000	33	10	1	49	11	0	12	76	11	109	Yes
Smith et al. [34]	2001	27	7	0	18	9	0	7	61	9	45	Yes
Smith et al. [34]	2001	17	3	0	18	9	0	3	37	9	45	Yes
Smith et al. [34]	2001	53	12	4	70	10	0	20	118	10	150	Yes
Smith et al. [34]	2001	27	4	0	52	12	1	4	58	14	116	Yes
Smith et al. [34]	2001	19	9	0	18	9	0	9	47	9	45	Yes
Smith et al. [34]	2001	20	6	0	70	10	0	6	46	10	150	Yes
Smith et al. [34]	2001	30	6	2	70	10	0	10	66	10	150	Yes
Smith et al. [34]	2001	29	11	0	52	12	1	11	69	14	116	Yes
Jiang et al. [21]	2005	37	19	3	221	66	4	25	93	74	508	Yes
Jiang et al. [21]	2005	173	61	8	221	66	4	77	407	74	508	Yes
Siezen et al. [24]	2006	155	42	4	307	79	4	50	352	87	693	Yes
Siezen et al. [24]	2006	380	92	7	555	162	9	106	852	180	1272	Yes
Kuriki et al. .[17]	2006	92		35^*^	117		61^*^					Yes
Kuriki et al. .[17]	2006	184		73^*^	543		226^*^					Yes
Wang et al. .[41]	2006	537	150	18	459	137	13	186	1224	163	1055	Yes
Vogel et al. [23]	2007	255	96	4	557	181	15	104	606	211	1295	Yes
Mossner et al. [42]	2007	242	73	20	273	73	7	113	557	87	619	Yes
Mossner et al. [42]	2007	377	113	7	316	111	8	127	867	127	743	Yes
Chang et al. [33]	2008	27	18	2	457	284	41	22	72	366	1198	Yes
Chang et al. [33]	2008	74	44	8	457	284	41	60	192	366	1198	Yes
Doecke et al. [38]	2008	190	65	5	1068	270	14	75	445	298	2406	Yes
Doecke et al. [38]	2008	223	72	6	1068	270	14	84	518	298	2406	Yes
Doecke et al. [38]	2008	170	41	2	1068	270	14	45	381	298	2406	Yes
Chang et al. [33]	2008	127	95	15	457	284	41	125	349	366	1198	Yes
Wu et al. [35]	2011	162	110	19	328	219	40	148	434	299	875	Yes
Wei et al. [37]	2013	115	69	15	122	69	9	99	299	87	313	Yes
Jeon et al. .[39]	2013	104	75	12	220	141	22	99	283	185	581	Yes
Park et al. [36]	2014	320	126	8	311	117	15	142	766	147	739	Yes
Our study	2017	297	198	26	631	355	43	250	792	441	1617	Yes

^*^Indicates TT+CT

HWE: Hardy–Weinberg equilibrium.

**Figure 2:**
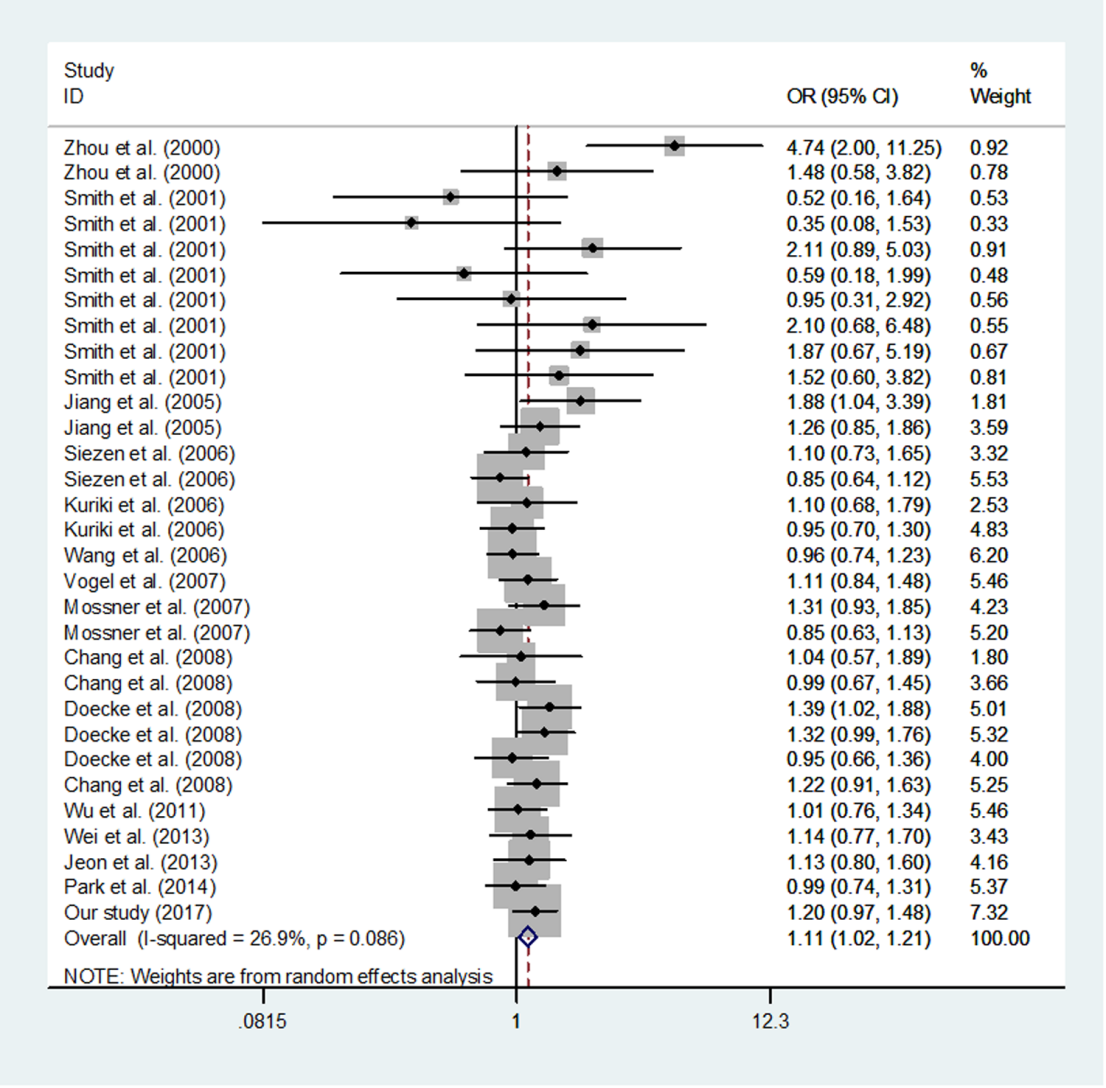
Meta-analysis of the association between *PPARG* c.1347C>T polymorphism and cancer risk (TT/CT vs. CC random–effects model).

Overall, we found a significant association between *PPARG* c.1347C>T polymorphism and the increased risk of cancer (T vs. C: OR, 1.13; 95% CI, 1.03–1.23; *P* = 0.006; TT vs. CC: OR, 1.29; 95% CI, 1.07–1.56; *P* = 0.008, CT/TT vs. CC: OR, 1.11; 95% CI, 1.02–1.21; *P* = 0.014 and TT vs. CT/CC: OR, 1.26; 95% CI, 1.04–1.52; *P* = 0.016; Table 6 and Figure 2).

**Table 6:** Results of the meta-analysis from different comparative genetic models

	No. of studies	T vs. C				TT vs. CC				TT+CT vs. CC				TT vs. CT+CC			
		OR(95% CI)	*P*	*I^2^*	*P*(Q-test)	OR(95% CI)	*P*	*I^2^*	*P*(Q-test)	OR(95% CI)	*P*	*I^2^*	*P*(Q-test)	OR(95% CI)	*P*	*I^2^*	*P*(Q-test)
Total	31	**1.13(1.03-1.23)**	**0.006**	39.5%	0.016	**1.29(1.07-1.56)**	**0.008**	6.6%	0.370	**1.11(1.02-1.21)**	**0.014**	26.9%	0.086	**1.26(1.04-1.52)**	**0.016**	1.9%	0.436
Ethnicity																	
Asians	15	**1.10(1.01-1.20)**	**0.033**	0.1%	0.444	1.19(0.94-1.51)	0.149	6.4%	0.382	1.10(1.00-1.21)	0.058	0.0%	0.704	1.15(0.91-1.45)	0.248	0.0%	0.447
Caucasians	9	1.14(0.91-1.43)	0.246	61.1%	0.008	1.33(0.87-2.03)	0.192	19.5%	0.275	1.14(0.89-1.45)	0.290	59.8%	0.011	1.32(0.87-2.02)	0.196	17.4%	0.293
Mixed	7	**1.26(1.02-1.55)**	**0.034**	50.5%	0.059	**1.70(1.08-2.68)**	**0.022**	1.7%	0.405	**1.17(1.01-1.35)**	**0.032**	34.3	0.166	**1.67(1.06-2.63)**	**0.027**	0.0%	0.462
Cancer type																	
Biliary tract cancer	3	1.10(0.92-1.31)	0.288	0.0%	0.729	1.21(0.76-1.93)	0.416	0.0%	0.848	1.12(0.90-1.38)	0.322	0.0%	0.675	1.17(0.74-1.84)	0.508	0.0%	0.865
Breast cancer	3	1.01(0.87-1.17)	0.890	0.0%	0.504	0.96(0.63-1.45)	0.843	47.8%	0.147	1.03(0.86-1.23)	0.785	0.0%	0.834	0.95(0.63-1.43)	0.810	48.1%	0.145
Colorectal cancer	7	1.10(0.97-1.25)	0.148	50.1%	0.143	1.48(0.95-2.30)	0.084	32.8%	0.299	1.06(0.94-1.21)	0.353	19.6%	0.370	1.45(0.93-2.25)	0.099	27.2%	0.345
Esophageal cancer	3	**1.22(1.04-1.44)**	**0.016**	33.7%	0.221	1.69(0.90-3.18)	0.102	0.0%	0.621	**1.23(1.03-1.47)**	**0.025**	27.7%	0.251	1.62(0.86-3.05)	0.131	0.0%	0.671
Glioblastoma	2	**2.54(1.43-4.54)**	**0.002**	53.8%	0.141	-	-	-	-	2.70(0.86-8.41)	0.087	68.4%	0.075	-	-	-	-
Melanoma	2	1.11(0.66-1.84)	0.698	84.5%	0.011	1.58(0.37-6.73)	0.539	78.4%	0.031	1.04(0.68-1.60)	0.849	72.2%	0.058	1.59(0.40-6.36)	0.514	76.5%	0.039
Ovarian cancer	3	1.01(0.55-1.86)	0.975	17.6%	0.297	-	-	-	-	1.05(0.54-2.02)	0.887	14.5%	0.311	-	-	-	-
Other cancers	8	1.11(0.99-1.25)	0.085	39.1%	0.118	1.35(0.96-1.91)	0.088	0.0%	0.471	1.11(0.96-1.28)	0.148	25.2%	0.228	1.29(0.92-1.82)	0.141	0.0%	0.476
The origin of cancer cell																	
Epithelial tumor	26	**1.11(1.04-1.19)**	**0.003**	20.5%	0.183	**1.25(1.01-1.54)**	**0.036**	0.0%	0.483	**1.11(1.03-1.20)**	**0.007**	0.0%	0.500	1.21(0.98-1.48)	0.074	0.0%	0.547
Non-epithelial tumor	5	1.29(0.91-1.83)	0.161	76.9%	0.002	1.54(0.96-2.46)	0.073	46.9%	0.130	1.27(0.87-1.86)	0.220	75.6%	0.003	1.55(0.97-2.47)	0.069	42.2%	0.158
Quality scores																	
≥7.0	19	**1.01(1.03-1.18)**	**0.005**	27.0%	0.146	**1.30(1.06-1.59)**	**0.012**	0.0%	0.540	**1.09(1.01-1.17)**	**0.021**	5.7%	0.386	**1.26(1.03-1.54)**	**0.025**	0.0%	0.614
<7.0	12	1.30(0.95-1.77)	0.099	54.4%	0.012	1.28(0.77-2.14)	0.348	38.8%	0.133	1.29(0.93-1.78)	0.129	48.7%	0.029	1.25(0.75-2.08)	0.388	37.7%	0.141

In a subgroup analysis by the ethnicity, evidence of significant association between *PPARG* c.1347C>T polymorphism and increased risk of cancer were also found among Asians, and mixed populations, but not Caucasians (Table 6). In a subgroup analysis by cancer type, c.1347C>T polymorphism was associated with the risk of esophageal cancer, and glioblastoma, but not biliary tract, breast, colorectal, melanoma, ovarian and other cancers (Table 6). In addition, in a subgroup analysis by the origin of cancer cell, evidence of significant association between *PPARG* c.1347C>T polymorphism and an increased risk of cancer were also found among epithelial tumor (Table 6).

The quality score of the enrolled studies was determined by using Newcastle-Ottawa Quality Assessment Scale [43].The results indicated that nineteen were high-quality and twelve were low-quality (Table 7). When we excluded the low-quality studies, the results were not substantially altered suggesting the reliability of our findings (Table 6).

**Table 7:** Quality assessment of the included studies in meta-analysis

Study	Year	Selection				Comparability of the cases and controls	Exposure			Total Stars
		Adequate case definition	Representativeness of the cases	Selection of the controls	Definition of Controls		Ascertainment of exposure	Same ascertainment method for cases and controls	Non-Response rate	
Zhou et al. [40]	2000	↔	↔	-	↔	-	↔	-	-	4
Zhou et al. [40]	2001	↔	↔	-	↔	-	↔	-	-	4
Smith et al. [34]	2001	-	-	-	↔	↔	-	-	-	2
Smith et al. [34]	2001	-	-	-	↔	↔	-	-	-	2
Smith et al. [34]	2001	-	-	-	↔	↔	-	-	-	2
Smith et al. [34]	2001	-	-	-	↔	↔	-	-	-	2
Smith et al. [34]	2001	-	-	-	↔	↔	-	-	-	2
Smith et al. [34]	2001	-	-	-	↔	↔	-	-	-	2
Smith et al. [34]	2001	-	-	-	↔	↔	-	-	-	2
Smith et al. [34]	2001	-	-	-	↔	↔	-	-	-	2
Jiang et al. [21]	2005	↔	↔	-	↔	↔↔	↔	↔	-	7
Jiang et al. [21]	2005	↔	↔	-	↔	↔↔	↔	↔	-	7
Siezen et al. [24]	2006	↔	↔	↔	↔	↔↔	↔	↔	-	8
Siezen et al. [24]	2006	↔	↔	↔	↔	↔↔	↔	↔	-	8
Kurikin et al. [17]	2006	↔	↔	-	↔	↔↔	↔	↔	↔	8
Kurikin et al. [17]	2006	↔	↔	-	↔	↔↔	↔	↔	-	7
Wang et al. [41]	2006	↔	↔	↔	↔	↔↔	↔	-	↔	8
Vogel et al. [23]	2007	↔	↔	↔	↔	↔↔	↔	↔	-	8
Mossner et al. [42]	2007	↔	↔	-	↔	↔↔	↔	↔	-	7
Mossner et al. [42]	2007	↔	↔	-	↔	↔↔	↔	↔	-	7
Chang et al. [39	2008	↔	↔	-	↔	↔↔	↔	↔	-	7
Chang et al. [39	2008	↔	↔	-	↔	↔↔	↔	↔	-	7
Chang et al. [39	2008	↔	↔	-	↔	↔↔	↔	↔	-	7
Doecke et al. [38]	2008	↔	↔	↔	↔	↔↔	↔	-	-	7
Doecke et al. [38]	2008	↔	↔	↔	↔	↔↔	↔	-	-	7
Doecke et al. [38]	2008	↔	↔	↔	↔	↔↔	↔	-	-	7
Wu et al. [35]	2011	↔	↔	-	↔	↔↔	↔	↔	-	7
Wei et al. [37]	2013	↔	-	-	↔	-	↔	-	-	3
Jeon et al. [39]	2013	-	↔	↔	↔	↔↔	↔	↔	-	7
Park et al. [36]	2014	↔	↔	-	-	↔↔	↔	↔	-	6
Our study	2017	↔	↔	-	↔	↔↔	↔	↔	-	7

**Figure 3:**
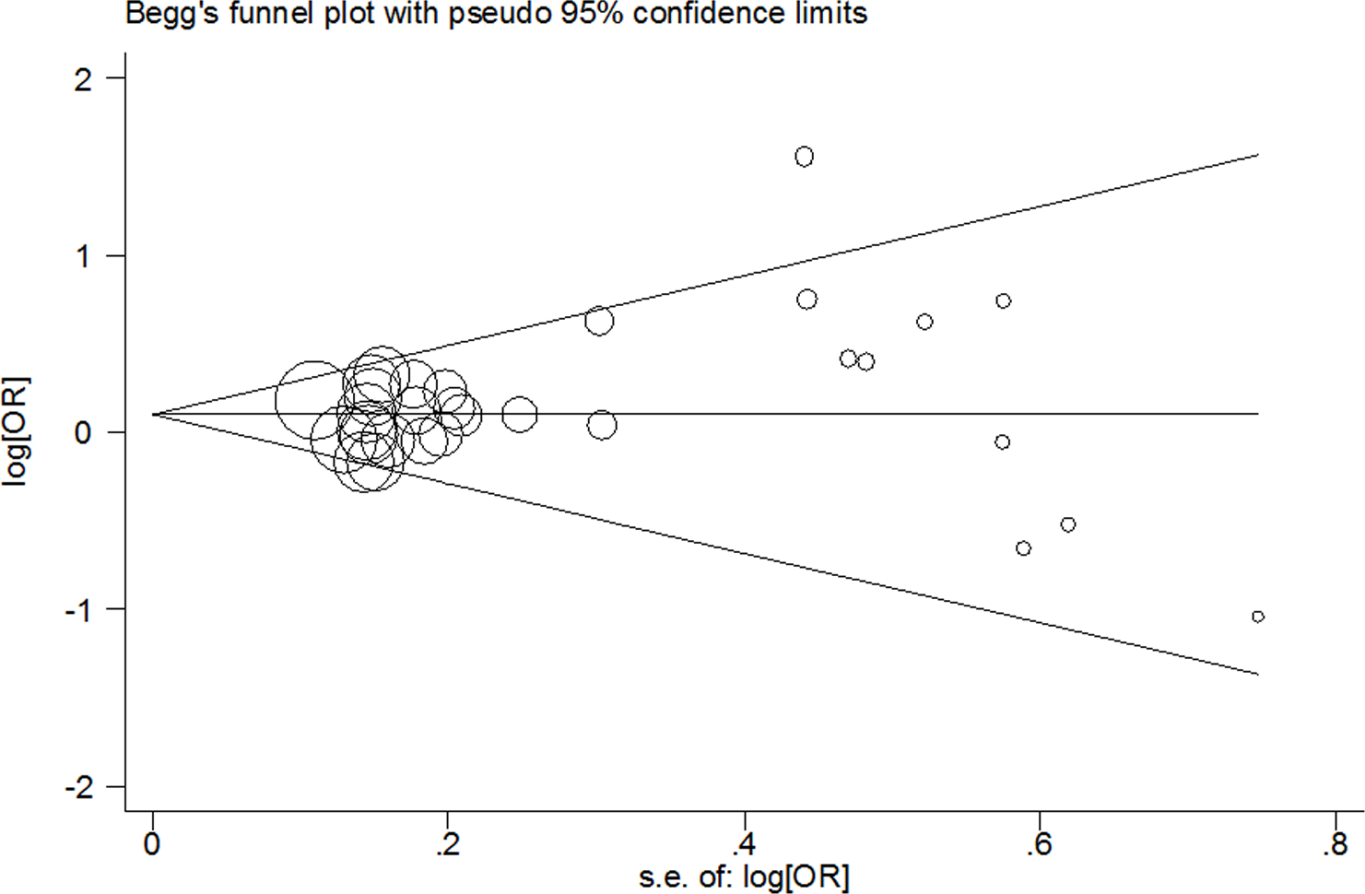
Begg's funnel plot of meta–analysis of the association between *PPARG* c.1347C>T polymorphism and cancer risk (TT/CT vs. CC compare genetic model, random–effects model)

**Figure 4:**
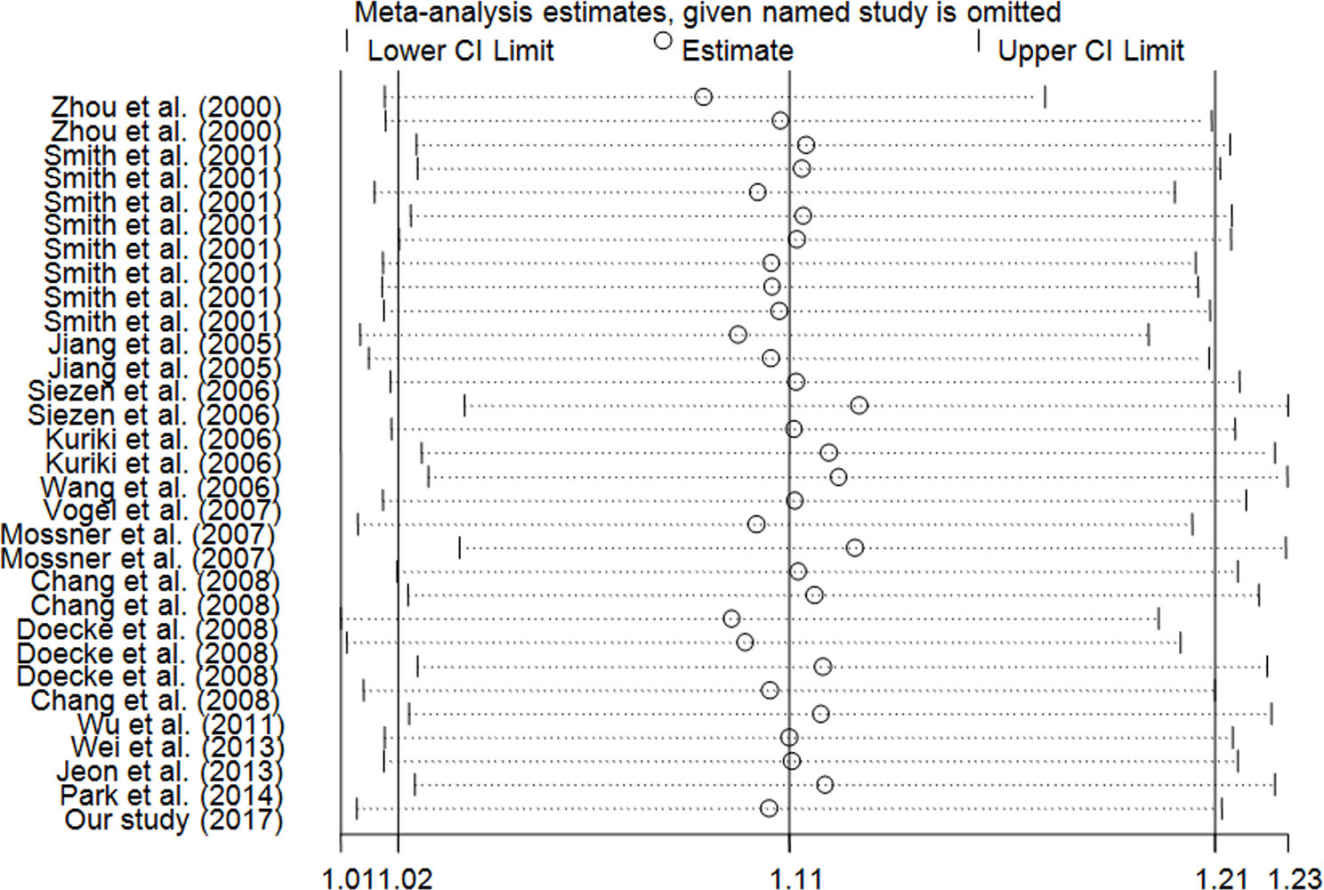
Sensitivity analysis of the influence of TT/CT vs. CC comparison (random–effects estimates for *PPARG* c.1347C > T polymorphism)

In this meta-anlysis, we used Begg's test and Egger's test to measure the publication bias. The results demonstrated that there was no significant bias in any genetic model (T vs. C: Begg's test *P* = 0.442, Egger's test *P* = 0.196; TT vs. CC: Begg's test *P* = 0.442, Egger's test *P* = 0.167; CT/TT vs. CC: Begg's test *P* = 0.634, Egger's test *P* = 0.244; TT vs. CT/CC: Begg's test *P* = 0.333, Egger's test *P* = 0.149; Figure 3). Using the one-way method (excluding an individual study in turn), sensitivity analysis was carried out to determine stability of our findings (Figure 4). The results indicated that our findings were stable and reliable.

Significant heterogeneities were found in this meta-analysis. Since the origin of cancer cell, quality score, ethnicity and cancer type could affect the results of meta–analysis, we carried out subgroup analyses by these factors and the findings were presented in Table 6. The results indicated that melanoma, non-epithelial tumor, Caucasians and quality score < 7.0 subgroups may contribute to the major heterogeneity. As shown in Table 6, significant heterogeneity was found in allele comparison, thus meta-regression was also performed to explore the source of heterogeneity. We found that quality score might contributed to major heterogeneity, which can explain 64.27% heterogeneity (Tau1 = 0.019100,Tau2 = 0.006824, respectively).

## DISCUSSION

The etiology of cancer was very complex. It is thought that many environmental and genetic factors may play important roles in the development of cancer. Multiple lines of evidence indicate a vital role for genetics in determining risk for cancer. PPARG is a member of the peroxisome proliferator-activated receptors (PPARs). PPARs interact with retinoid X receptors and then regulate the transcription process of many genes. PPARG has been implicated in the development of various diseases involving obesity, diabetes, inflammation, atherosclerosis and cancer [44–47]. PPARG is expressed in various cancer cells. There are accumulating evidences that obesity/overweight, type 2 diabetes, inflammation, and malignancy are etiologically related [48, 49]. Being at the crossroads of multiple diseases, PPARG may be a key component for understanding the pathophysiology of cancer. In this study, we explored the relationship of *PPARG* c.1347C>T polymorphism with NSCLC risk. Then, we conducted a comprehensive meta-analysis to further understand the potential role of this SNP for the susceptibility to overall cancer. In the case-control study, we found an association between *PPARG* c.1347C>T polymorphism and a tendency to increased risk of NSCLC. Along with a meta-analysis, we found that *PPARG* c.1347C>T polymorphism was associated with the increased risk of overall cancer. To the best of our knowledge, this study is the first case-control study focusing on the association between *PPARG* c.1347C>T polymorphism and NSCLC risk in Asians. And we first confirmed the relationship between this SNP and overall cancer risk.

With the increasing studies on genetic association, it is necessary to analyze the available data to obtain robust, replicable results. Considering the fact that a common SNP may make a small-to-moderate contribution to the risk of cancer, this pooled-analysis urges the necessity of adequate sample sizes to get a precise measurement between *PPARG* c.1347C>T polymorphism and the development of cancer. Several individual studies have reported positive signals of *PPARG* c.1347C>T polymorphism with cancer risk [21, 38, 40]; however, others observed null association. Recently, a meta-analysis reported that this polymorphism was not associated with cancer risk [31]; however, this pooled-analysis only included four case-control studies. In this updated meta-analysis, overall findings among 21,404 subjects, evidence of significant association between this polymorphism and cancer risk were found, even in Asians, mixed populations, esophageal cancer, glioblastoma and epithelial tumor subgroups. In *PPARG* exon 6, a C to T substitution is a synonymous polymorphism which encodes histidine either with *PPARG* c.1347 C or T allele. The findings of previous epidemiological studies showed a relationship of this polymorphism with metabolic diseases such as type 2 diabetes and atherosclerosis [50–53]. It is proposed that the C to T substitution may modulate the expression of PPARG by altering mRNA processing or translation. A tendency of increased risk was observed for *PPARG* c.1347C>T polymorphism with NSCLC risk, and an increased risk was also found in the subsequent meta-analysis. These consistent findings demonstrated that *PPARG* c.1347C>TC>T polymorphism might influence the development of cancer. In the future, further evaluations with detailed environmental factors are warranted to confirm these results.

Additionally, some potential limitations should be further addressed when interpreting our findings. First, the design of our case-control study was hospital-based, and the selecting bias might have occurred. Second, in this meta-analysis, the included studies based on the published studies, unpublished articles might fail to be retrieved. Third, since the significant heterogeneities were found in this meta-analysis, our findings should be interpreted with cautions. Fourth, lack of the data on environmental factors (e.g. lifestyle, fasting plasma glucose, total cholesterol, high-density lipoprotein cholesterol, low-density lipoprotein cholesterol, serum triglycerides etc.), the corresponding subgroup analyses were not conducted. Finally, we only focused on c.1347C>T polymorphism in *PPARG* gene, and did not consider other susceptibility genes or polymorphisms.

In conclusion, this case-control study in Eastern Chinese Han populations, along with a comprehensive meta-analysis, identify the association of *PPARG* c.1347C>T polymorphism with an increased risk of cancer, even in Asians, esophageal cancer, glioblastoma and epithelial tumor subgroups. Nevertheless, for some practical reasons, we hope that more case-control studies with the detailed environmental data to further explore the molecular mechanism of *PPARG* c.1347C>T polymorphism with development of cancer.

## MATERIALS AND METHODS

### Subjects

Genotyping analyses were carried out on genomic DNA of 521 NSCLC patients and 1,030 unrelated controls. All participants were come from Eastern Chinese Han population. The major included criterion of NSCLC patients were: (A) living in Eastern China area; (B) NSCLC was confirmed by pathological examination; (C) without autoimmune disease. The NSCLC patients comprised unrelated subjects who had been treated in Affiliated People's Hospital of Jiangsu University and Fujian Medical University Union Hospital. The blood samples were collected from January 2014 to December 2016. Index cases were first diagnosed with NSCLC. All patients gave a written informed consent.

The controls included healthy blood donors collected in the same hospitals, having the same ethnic background and similar lifestyle as the NSCLC patients. The controls were biologically unrelated to the NSCLC cases and were cancer-free. The age distribution of NSCLC cases and non-cancer controls was nearly identical (controls: 60.34 ± 9.11 years; cases: 59.76 ± 10.71years; *P* = 0.268). The sex distribution of NSCLC cases and controls was well-matched (*P* = 0.453). According to the guidelines of Chinese blood donation, each participant was examined by a questionnaire and wrote his/her informed consent. The controls were randomly collected during the years 2014–2016. The study was approved by the Ethics Committee of Jiangsu University (Zhenjiang, China) and Fujian Medical University (Fuzhou, China).

### DNA extraction and genotyping

EDTA anticoagulant vacutainer tube was used to collect blood sample. We used DNA Kit (Promega, Madison, USA) to extract the genomic DNA from the whole blood.

*PPARG* c.1347C>T polymorphism (NP_005028.4: p.His449His) was analyzed using SNPscan^TM^ genotyping assay (Genesky Biotechologies Inc., Shanghai, China). The SNP assays were confirmed by re-genotyping sixty-two (4%) randomly selected samples.

### Statistical analysis

The continuous variables (e.g. age, and BMI) are presented as the mean ± SD. We used Student's *t*-test to examine the difference of continuous variables between NSCLC patients and non-cancer controls. In addition, we used *χ*^2^ test to determine the difference of categorical variables (e.g. genotypes, smoking status, alcohol consumption, sex, age and BMI). HWE test in controls was undertaken using an internet-based *χ*^2^ goodness-of-fit test (http://ihg.gsf.de/cgi-bin/hw/hwa1.pl). Genotype-specific ORs with their corresponding 95%CIs and *P*-values were calculated by SAS 9.4 software for windows (SAS Institute, Cary, NC). *P*-values were presented using two-sided *χ*^2^-test.

### Meta-analysis

To further determine the relationship between *PPARG* c.1347C>T variants and cancer susceptibility, we carried out a meta-analysis. All studies focusing on the association between this polymorphism and cancer risk were collected by searching of PubMed and Embase databases (the last search update on June 12, 2017). The search was performed with the terms of (Peroxisome proliferator activated receptor gamma or PPARG) and (NP_005028.4: p.His449His or His449His or H449H or C161T or C1431T or rs3856806 or c.1347C>T) and (polymorphism or variant) and (cancer or carcinoma). Additional studies were also supplemented by a hand search of the corresponding references in retrieved articles. In this study, the language of publication was restricted to English. In our analysis, eligible studies had to meet the inclusion criteria: (1) focusing on the association between *PPARG* c.1347C>T polymorphism and cancer risk; (2) designed as a case-control or cohort study; (3) data could be extracted from the publications (genotypes of cases and controls); (4) published in English language; (5) genotype distribution was consistent with HWE in controls. Two authors (H. Ding and H. Qiu) extracted the detailed information from the eligible publications independently. When they met the disagreement, the third reviewer (Y. Chen) was invited to discuss every item. Finally, a consensus was reached. The following characteristics were selected and collected: the first author, year, country, ethnicity, genotyping method, cancer type, sample size, the origin of cancer cell and genotype frequencies.

For each included study, we analyzed HWE in controls using goodness-of-fit test mentioned above and *P* < 0.05 was defined as violation of HWE. Crude ORs with their 95% CIs were used to examine the strength of relationship between *PPARG* c.1347C>T polymorphism and cancer susceptibility. The pooled ORs for this polymorphism were performed under four genetic models (e.g. TT+CT vs. CC, TT vs. CC+CT, TT vs. CC and T vs. C). Stratified analyses were extensively performed with respect to origin of cancer cell, ethnicity, cancer type and quality scores. The heterogeneity across the eligible studies was tested by using a χ^2^-based Q-test and *I^2^* test [54]. The pooled OR was calculated by a random-effects model (the Der-Simonian and Laird method) if *I^2^* > 50% or *P* < 0.1, which indicated that heterogeneity was significant [55, 56]. Otherwise, the pooled OR was assessed by a fixed-effects model (the Mantel-Haenszel method) [57]. Removing each study in turn, sensitivity analysis was carried out by one-way method to determine the stability of the results. Additionally, Begg's test and Egger's linear regression test were conducted to assess the potential publication bias [58] and *P* < 0.1 was regarded as a bias. Meta-regression was conducted to analyze the source of heterogeneity [59]. In the present meta-analysis, all statistical analyses were performed by using the STATA 12.0 software for windows (Stata Corporation, College Station, Texas). A *P* value (two-sided) less than 0.05 were considered significant. Newcastle-Ottawa Quality Assessment Scale was harnessed to determine the quality score of the enrolled studies. If scores ≥ 7 stars, the study was defined as high-quality [43, 60].
